# Phylogenetic and mutational analysis of H10N3 avian influenza A virus in China: potential threats to human health

**DOI:** 10.3389/fcimb.2024.1433661

**Published:** 2024-06-24

**Authors:** Jingyi Dai, Jun Zhao, Jiawei Xia, Pei Zhang, Yadi Ding, Qiujing Li, Min Hou, Xianhui Xiong, Qianqi Jian, Yanyan Liu, Guiming Liu

**Affiliations:** ^1^ Department of Public Laboratory, The Third People's Hospital of Kunming City/Infectious Disease Clinical Medical Center of Yunnan Province, Kunming, Yunnan, China; ^2^ School of Public Health, Hubei University of Medicine, Shiyan, China; ^3^ Department of Microbiological Laboratory, Kunming City Center for Disease Control and Prevention, Kunming, China

**Keywords:** H10N3, avian influenza A virus, human infection, phylogeny analysis, mutation

## Abstract

In recent years, the avian influenza virus has emerged as a significant threat to both human and public health. This study focuses on a patient infected with the H10N3 subtype of avian influenza virus, admitted to the Third People’s Hospital of Kunming City on March 6, 2024. Metagenomic RNA sequencing and polymerase chain reaction (PCR) analysis were conducted on the patient’s sputum, confirming the H10N3 infection. The patient presented severe pneumonia symptoms such as fever, expectoration, chest tightness, shortness of breath, and cough. Phylogenetic analysis of the Haemagglutinin (HA) and neuraminidase (NA) genes of the virus showed that the virus was most closely related to a case of human infection with the H10N3 subtype of avian influenza virus found in Zhejiang Province, China. Analysis of amino acid mutation sites identified four mutations potentially hazardous to human health. Consequently, this underscores the importance of continuous and vigilant monitoring of the dynamics surrounding the H10N3 subtype of avian influenza virus, utilizing advanced genomic surveillance techniques.

## Introduction

With the continuous advancement of urbanization and rural development, the frequency and scope of contact between humans and animals are constantly expanding (Schell et al., 2021). In cities, people have close contact with poultry and pets, while in rural areas, farmers and breeders deal directly with poultry and livestock. This kind of contact is not limited to daily life, but also includes activities such as poultry raising, breeding, slaughtering, and other activities, providing opportunities for the spread of pathogens (Tobin et al., 2015; Moyen et al., 2021). Moreover, increased global trade and travel have intensified the risk of cross-border disease transmission (Shah et al., 2019). The international trade of animals and their products facilitates the rapid spread of pathogens worldwide. Simultaneously, human transnational travel creates favorable conditions for pathogen dissemination. The occurrence of a single case can rapidly attract global attention and vigilance. In this era of interconnectedness and swift information dissemination, avian influenza, as a significant zoonotic viral infectious disease, has garnered considerable concern due to its potential transmission risks and hazards. Consequently, enhancing surveillance, prevention, and control measures for zoonotic diseases like avian influenza has emerged as a crucial global public health priority.

Avian influenza virus is a type of RNA virus, classified under the genus Influenza A virus of the family Orthomyxoviridae, and is categorized into types A, B, C, and D (Hu et al., 2018). Among these, avian influenza A virus is particularly concerning for human health, as it can lead to severe illness and even fatalities. Avian influenza virus is polymorphic, with a spherical diameter ranging from 80 to 120 nm and possessing an envelope. Its genome consists of segmented single-stranded negative-sense RNA, making it highly variable (Spackman, 2008). Based on the antigenicity of its hemagglutinin (HA) and neuraminidase (NA) proteins on the outer membrane, it is currently classified into 18 H subtypes (H1-H18) and 11 N subtypes (N1-N11) (Li et al., 2021). Human transmission of avian influenza viruses usually occurs through contact with infected poultry or their excreta, secretions, etc., particularly in settings such as poultry farming, slaughtering, and handling, where the risks are heightened (Wong and Yuen, 2006). Individuals infected with avian influenza virus may display a range of symptoms, with severity varying among cases (Horman et al., 2018). Common symptoms include fever, cough, runny nose, headache, muscle and joint pain, fatigue, breathing difficulties, chest tightness, respiratory failure, nausea, vomiting, diarrhea, etc. Infection with this virus can lead to severe complications like pneumonia and acute respiratory distress syndrome (ARDS), especially in individuals with underlying health conditions or weakened immune systems. Therefore, prompt medical attention is essential when experiencing such symptoms, especially if severe manifestations like breathing difficulties arise. To date, confirmed subtypes of avian influenza A viruses that have infected humans include H6N1, H9N2, H10N8, H5N6, H7N4, H10N3, and H5N8, etc (Yang et al., 2022). Patients infected with H5N1, in particular, often experience severe illness with a high mortality rate.

Currently, avian influenza virus infection cannot be diagnosed based on clinical manifestations alone; instead, laboratory testing is required (Geyer et al., 2022). Commonly used laboratory testing methods include serological diagnostic methods such as hemagglutination and hemagglutination inhibition tests, neuraminidase inhibition test, agarose diffusion test, and enzyme-linked immunosorbent assay. Additionally, molecular biology diagnostic technologies such as reverse transcription PCR (RT-PCR), fluorescent RT-PCR, and Next Generation Sequencing (NGS) are employed. NGS technology, in particular, provides a powerful tool for the detection and research of avian influenza viruses (Soda et al., 2023). It allows high-throughput and deep sequencing of viral genomes, providing detailed genomic information, including the virus’s full genome sequence, mutations, recombination events and gene expression levels (Quer et al., 2022). This technology not only enables highly sensitive and specific detection, but also aids researchers in understanding virus evolution, transmission, and host interactions.

In recent years, human infection with avian influenza virus has been frequently reported around the world, especially involving subtypes H5, H7, and other (Gao, 2018; Liang, 2023; Szablewski et al., 2023). Among these, human infections with H10 avian influenza A virus have been reported globally, including subtypes H10N7, H10N8, and H10N3 (Arzey et al., 2012; Chen et al., 2014). The H10N3 subtype of avian influenza A virus has been circulating among waterfowl and poultry in East and South Asia for decades, with rare instances of human infection (Wisedchanwet et al., 2011). The first recorded human cases of Avian-Origin Influenza A (H10N3) virus occurred in Jiangsu, China, in April 2021 (Qi et al., 2022), followed by a second case reported in Zhejiang in June 2022 (Zhang et al., 2023).

In this study, we utilized metagenomic NGS (mNGS) and nanopore metagenomic sequencing to document the first recorded instance of human infection with avian influenza A virus H10N3 in Yunnan Province. This case marks the occurrence of H10N3 human infection in China. To elucidate the virus’s origin, we conducted a comprehensive epidemiological investigation, performed phylogenetic analysis, and aligned the virus’s entire genome sequence with homologous sequences. Additionally, to evaluate the virus’s adaptability and pathogenicity in human hosts, we analyzed the amino acid mutation sites of three H10N3 subtype AIV infections among humans.

## Materials and methods

### Data collection

On March 6, 2024 the patient went to Kunming Third People’s Hospital for treatment due to persistent fever for many days. The diagnosis revealed severe pneumonia, type I respiratory failure, and infection with avian influenza A virus. After the diagnosis of avian influenza A virus infection, the patient underwent investigation through questionnaires, which included demographic information, poultry contact history, underlying health conditions, and other relevant data.

### Genomic analysis and genome assembly

Multiple amplification products were obtained using influenza A virus genotyping gene targeted amplification kit (BaiyiTech, Hangzhou). The amplified products were purified using ampure XP beads nucleic acid magnetic bead Purification Kit (Beckman, USA) and the library was constructed. The library was constructed by ligation method with the kit sqk-nbd114.24 (Nanopore, UK). After the library was constructed, it was added to the flo-min 114 sequencing chip (Nanopore, UK), and high-throughput sequencing was performed on the gridion X5 third-generation sequencer. All experimental procedures were meticulously performed in strict accordance with the instructions provided by the respective kits and the requirements for the nanopore third-generation high-throughput sequencing.

### Phylogenetic analysis

The nucleotide sequences obtained were analyzed in the Genbank and GISAID databases (www.gisaid.org) using Nucleotide Basic Local Alignment Search Tool (BLAST) software of NCBI to initially determine the virus subtypes. Similar HA and NA nucleotide sequences were downloaded for phylogenetic analysis. The nucleotide and amino acid sequences were aligned using MAFFT (v7.310), and the phylogenetic trees was constructed based on the neighbor-joining method using MEGA-X.

### Ethics statement

The patient and his family members signed consent forms approving the investigation, sample collection and its publication. The procedures were in accordance with the Helsinki declaration of 1975, as revised in 1983. This study was approved by the Medical Ethics Committee at Kunming hird People’s Hospital (No. KSLL20230711009) and adhered to the guidelines established in the Declaration of Helsinki. To protect patients’ personal information, including names and ID numbers, it was encrypted before use.

## Results

A previously healthy 51-year-old male experienced recurrent fever for a week, reaching a maximum temperature of 39°C, accompanied by symptoms of cough, expectoration, chest tightness, and shortness of breath. Despite seeking medical attention at the local community health service center, his symptoms did not significantly improve. Consequently, he was transferred to the Department of Respiratory and Critical Care Medicine at the Third People’s Hospital of Kunming City in Yunnan Province, Southwest China, on March 6, 2024.

**Figure 1:**
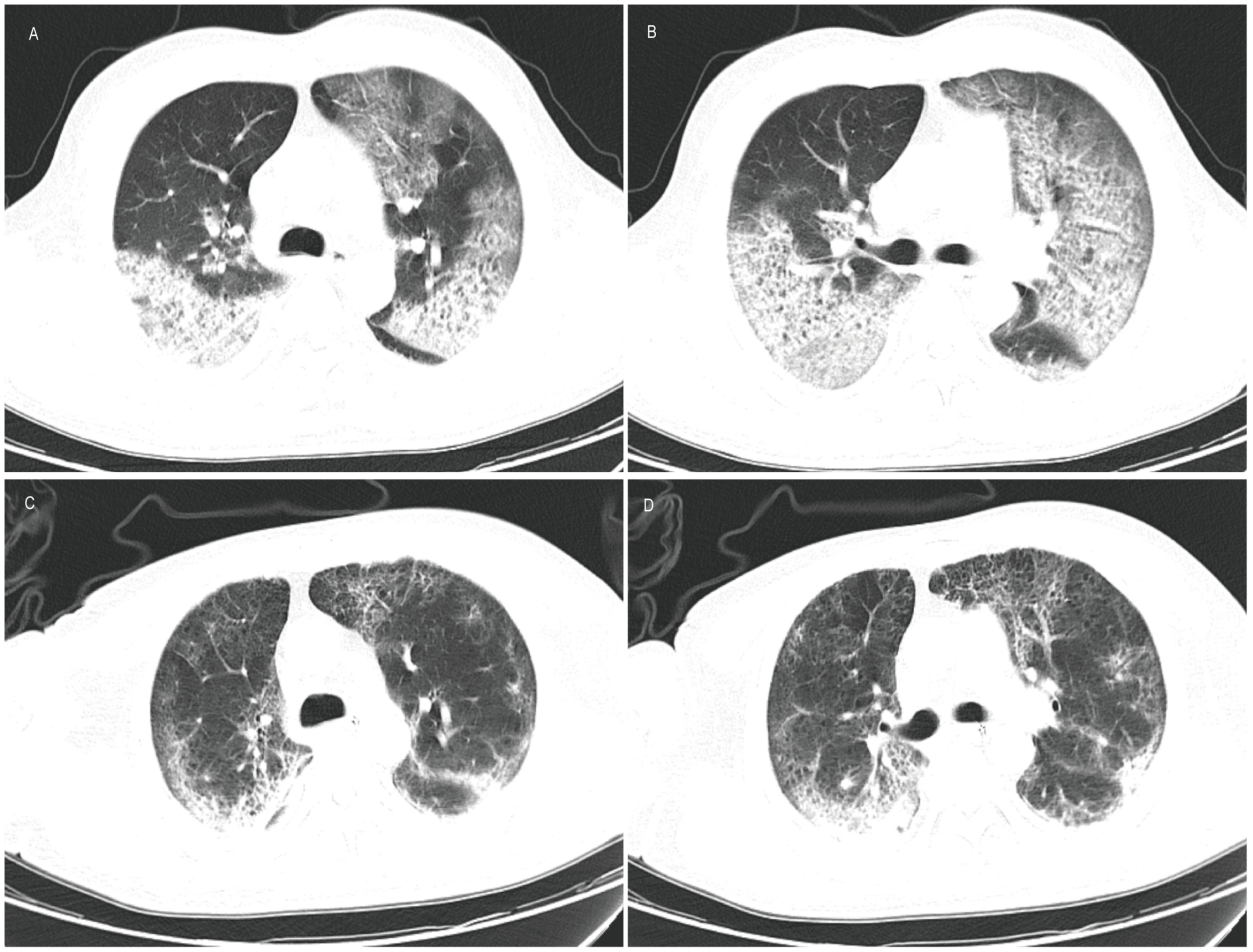
Computed tomography of lung. **(A, B)** Results on March 6, 2024 showed that multiple patchy and patchy increased density shadows were seen in both lungs, with unclear boundary and uneven density; **(C, D)** Results on March 23, 2024 showed a reduction in lesions compared to previous scans.

Upon admission (7 days after the onset of illness), the patient presented with a temperature of 39℃, a pulse rate of 110 beats per minute, a respiratory rate of 28 breaths per minute, oxygen saturation of 78%, and blood pressure measuring 105/70 mmHg (**Table 1**). Laboratory tests revealed a low white blood cell count, elevated neutrophil percentage, decreased platelet count, and elevated levels of infectious markers. Additionally, a throat swab specimen tested positive for influenza A virus nucleic acid by PCR (**Table 2**). Chest computed tomography (CT) revealed multiple patchy and increased density shadows in both lungs, characterized by unclear boundaries and uneven density (**Figure 1**). The initial diagnosis upon admission included severe pneumonia, type I respiratory failure, and influenza attributed to influenza A virus.

**Table 1:** Patient’s characteristics and clinical symptoms.

Characteristic/Symptom	Value
Age (years)	51
Sex	Male
Date of illness onset	29 Feb 2024
Date of admission	6 Mar 2024
Date of discharge	17 Apr 2024
Signs or symptoms	
Fever	Yes
Body temperature (°C)	39.0
Cough	Yes
Sputum production	Yes
Dizzy	Yes
Weakness	Yes
Chest tightness	Yes
Bacterial culture	Staphylococcus epidermidis, Acinetobacter joni, Carbapenem-resistant Enterobacter cloacae
Fungi culture	Candida albicans
Glucocorticoid therapy	Yes
Antibiotic therapy	Meropenem, omacycline, voriconazole, levofloxacin, amikacin
Antiviral therapy	Oseltamivir
Anticoagulant therapy	Low molecular weight heparin calcium
Oxygen therapy	Noninvasive ventilator positive pressure ventilation

**Table 2:** Laboratory Test Results.

Indicators	Day 7 (6 Mar)	Day 22 (21 Mar)	Normal range
White Blood Cell (×10^9^cells/L)	2.12	8.58	3.50–9.50
Neutrophil (×10^9^cells/L)	1.80	7.19	1.80–6.30
Neutrophil percentage (%)	84.90	83.90	40.00–75.00
Lymphocyte (×10^9^cells/L)	0.26	0.74	1.10–3.20
Lymphocyte percentage (%)	12.30	8.60	20.00–50.00
Blood platelet (×10^9^cells/L)	79	223	125–350
Prothrombin time (s)	15.9	16.6	14.0–16.0
Hypersensitive C-reactive protein (mg/L)	249.41	21.93	0.00–6.00
Lnterleukin-6 (pg/mL)	78.99	8.26	0.00–7.00
Procalcitonin (ng/mL)	14.040	0.248	<0.500
pO_2_ (mmHg)	32.00	68.40	80.00–100.00
pCO_2_ (mmHg)	32.00	52.10	35.00–45.00
Nucleic acid testing for influenza A virus	Positive	Negative	Negative

The patient was administered oseltamivir (150mg, twice daily) and methylprednisolone (80mg, once daily) and corresponding antibiotics for treatment. Subsequent sputum culture results revealed infection with *Candida albicans* and *Staphylococcus epidermidis* and *Acinetobacter joni* and Carbapenem-resistant *Enterobacter cloacae*, prompting the administration of appropriate antibiotics (**Table 1**). The patient’s fever subsided on March 17th (18 days after illness onset), and on March 19th (20 days after illness onset), the nucleic acid test for influenza A virus returned negative results for the first time. Subsequent test results on March 21st (22 days after illness onset) indicated normalization of the patient’s white blood cell count, along with a decrease or return to normal levels of infection markers. However, the patient exhibited prolonged prothrombin time. Chest computed tomography scans showed a reduction in lesions compared to previous scans (**Figure 1**). The lung lesions were noticeably absorbed, and there was no chest tightness or dyspnea. The patient was discharged on April 17th, and home oxygen therapy was recommended.

Although the nucleic acid detection of influenza A virus confirmed the patient’s infection with influenza A virus, it could not determine the specific subtype. A sputum sample collected from the patient on March 8 was confirmed as H10N3 subtype by mNGS and PCR in the Third People’s Hospital of Kunming City and the Kunming City Center for Disease Control and Prevention (CDC). However, epidemiological investigations revealed that the patient had a history of raising various birds, including chickens, ducks, geese, pigeons, peacocks, and ostriches. Notably, more than 20 chickens and geese died in the week preceding the onset of his illness, and he had a history of slaughtering these birds. However, the virus has not been detected in the environment or in poultry carcasses. No avian influenza A virus infection was detected in his close contacts.

**Figure 2:**
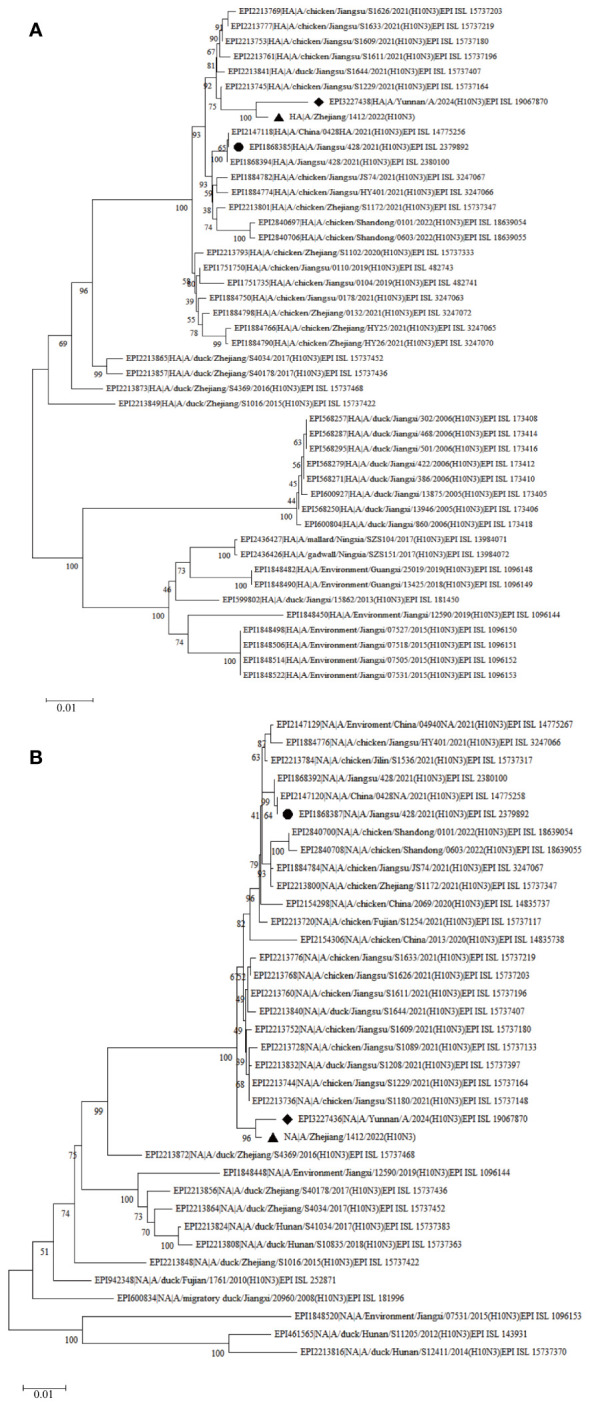
Phylogenetic trees of H10N3 strains based on nucleotide sequence. **(A)** Phylogenetic tree of HA; **(B)** Phylogenetic tree of NA. The Phylogenetic trees were downloaded from the GISAID database (https://gisaid.org) using the neighbor-joining method in MEGA X. The diamond indicates the H10N3 strain in this study, and the octagon indicates the H10N3 strain from the first case in Jiangsu.

To obtain the complete genome sequence of the virus and clarify its molecular characteristics, the Kunming City CDC conducted nanopore sequencing (Nanopore, GridION X5) on the samples, resulting in the acquisition of the whole genome information of the samples (GISAID#EPIISL19067870), named A/Yunnan/A/2024(H10N3). Online analysis using BLASTN software on the GISAID website revealed that all eight gene segments of the H10N3 virus strain in our case originated from Eurasian avian influenza viruses. Analysis of the evolutionary trees of the Haemagglutinin (HA) and neuraminidase (NA) genes suggested that the patient’s strain is a cross-species infection of the H10N3 virus, which has been prevalent in poultry in China in recent years (**Figure 2**). Homologous comparison in GenBank with BLAST revealed a high similarity between Yunnan strain’s HA gene (98.64%) and NA gene (99.43%) with A/Zhejiang/1412/2022(H10N3) virus (**Table 3**). Further analysis of amino acid mutation sites revealed a mutation at the 226th amino acid residue in the receptor binding site of the HA protein, where the amino acid changed from Q to L. Additionally, key mutations were identified, including D701N of PB2 protein, S409N of PA protein, and S31N of M2 protein (**Table 4**).

**Table 3:** Sequence similarity comparison, A/Yunnan/A/2024(H10N3) compared with A/Zhejiang/1412/2022(H10N3) (ZJ1412) and A/Jiangsu/428/2021(H10N3) (JS428).

Description	Query cover	Per.Ident	ACC.LEN	Description	Query cover	Per.Ident	ACC.LEN
PB2				NP			
ZJ1412	97%	99.12%	2280	ZJ1412	96%	99.47%	1497
JS428	99%	96.67%	2280	JS428	100%	96.19%	1497
PB1				NA			
ZJ1412	98%	99.65%	2274	ZJ1412	98%	99.43%	1410
JS428	99%	95.78%	2274	JS428	100%	98.01%	1410
PA				MP			
ZJ1412	97%	99.44%	2151	ZJ1412	95%	99.49%	982
JS428	96%	97.21%	2151	JS428	95%	97.56%	982
HA				NS			
ZJ1412	99%	98.64%	1686	ZJ1412	97%	99.39%	838
JS428	100%	97.27%	1686	JS428	94%	96.66%	838

**Table 4:** Mutations in A/Yunnan/A/2024(H10N3) and JS428 and ZJ1412, by gene.

	Biological function	Mutation	Yunnan /A	Jiangsu /428	Zhejiang /1412
HA	Receptor binding sites	Q226L	L	Q	Q
		G228S	G	S	G
		R229I	R	I	R
	Cleavage site		PEIIQGR↓G	PEIIQGR↓G	PEIIQGR↓G
NA	Antiviral resistance	E119V	E	E	E
		Q136K	Q	Q	Q
		I222M	I	I	I
		R292K	R	R	R
		R371K	R	R	R
PB2	Mammalian adaptation	Q591K	Q	K	Q
		E627K	E	E	E
		D701N	N	D	N
PB1	Increased transmission in ferret	I368V	V	V	V
PA	Host signature	V100A	V	V	V
		S409N	N	N	N
M2	Antiviral resistance	S31N	N	N	N
NS1	Increased virulence in mice	D92E	D	D	D
		P42S	P	S	S

## Discussion

Avian influenza virus is typically known for its strong host species preference and limited transmission to other species (Kim et al., 2021). However, due to the 8-segment nature of the viral genome and the RNA error replication mechanism, frequent recombination and mutation of the virus can occur, potentially enabling it to survive and spread in other species (Kim et al., 2021). As a result, various strains of avian influenza viruses have been identified in marine mammals, terrestrial poultry, horses, dogs, pigs, and notably, humans (Lloren et al., 2017; Kim et al., 2021; Kok et al., 2023).

The patient in Yunnan not only exhibited infection with the H10N3 subtype of influenza A virus but also presented with a mixed infection involving drug-resistance bacteria and fungi, making the condition complex. It’s worth noting that severe pneumonia patients infected with avian influenza often experience concurrent or secondary bacterial and fungal infections (Chow et al., 2019). Therefore, it is recommended to conduct repeated sputum culture, respiratory tract aspirate culture, or mNGS detection in clinical settings to identify the types of bacteria or fungi present, as well as their susceptibility or resistance patterns. This approach enables clinicians to make informed decisions regarding antibiotic selection and guide appropriate clinical treatment strategies.

The clinical manifestations of avian influenza A virus infection vary depending on the virus subtypes involved. For instance, infection with H5N1 and H7N9 subtypes can lead to severe pneumonia and related complications in patients. Conversely, certain subtypes such as H7 and H9 may only induce conjunctivitis or mild respiratory symptoms (Liu et al., 2013). It’s important for healthcare providers to be aware of these differences in clinical presentation when diagnosing and managing cases of avian influenza virus infection. As of now, only two cases of human infection with the H10N3 subtype have been reported. The symptoms observed in the patient infected with H10N3 in this case closely resemble those documented in the two previously known cases of H10N3 infection. Notably, all cases resulted in severe pneumonia in the affected patients (Qi et al., 2022; Zhang et al., 2023).

However, the molecular features of these cases are different, and our case has some different mutations. The Q226L mutation makes the virus more adept at binding to human α-2,6-sialic acid receptors, significantly increasing the likelihood of human infection (Shi et al., 2014). The mutation D701N in the PB2 protein has been shown to enhance the replication activity of avian influenza RNA polymerase within the human body. This mutation also increases the adaptability and pathogenicity of the virus to the human host, potentially serving as a crucial factor in avian influenza viruses crossing the host species barrier (Li et al., 2005). The presence of the S409N mutation in the PA protein suggests the potential for infectivity in humans and may contribute to increased pathogenicity of this particular virus strain (Finkelstein et al., 2007). The S31N mutation in the M2 protein has been associated with resistance to adamantanes, a class of antiviral drugs (Pielak et al., 2009). This mutations in the protein of the Yunnan H10N3 virus strain underscores the potential for increased threat posed by H10N3 in humans. Therefore, it is imperative to closely monitor the dynamics of this subtype.

The case of human infection with H10N3 avian influenza A virus highlighted in this study involved close contact with live birds, particularly through the handling and slaughtering of dead birds. Although there is no direct evidence, it is likely that this exposure eventually resulted in the patient contracting avian influenza and experiencing severe illness. This underscores the importance of paying special attention to instances of unexpected bird deaths and promptly reporting such cases. Moreover, it emphasizes the necessity of establishing a comprehensive avian influenza surveillance system, not only within Yunnan but also globally, to continuously and vigilantly monitor the H10N3 virus strain and its potential impact on human health.

## Data availability statement

The datasets presented in this study can be found in online repositories. The names of the repository/repositories and accession number(s) can be found in the article/supplementary material.

## Ethics statement

The studies involving humans were approved by Medical Ethics Committee at Kunming Third People’s Hospital. The studies were conducted in accordance with the local legislation and institutional requirements. The human samples used in this study were acquired from a by- product of routine care or industry. Written informed consent for participation was not required from the participants or the participants’ legal guardians/next of kin in accordance with the national legislation and institutional requirements. Written informed consent was obtained from the individual(s) for the publication of any potentially identifiable images or data included in this article.

## Author contributions

JD: Conceptualization, Data curation, Supervision, Writing – original draft, Writing – review & editing. JZ: Conceptualization, Formal analysis, Software, Supervision, Writing – original draft, Writing – review & editing. JX: Conceptualization, Resources, Supervision, Writing – original draft, Writing – review & editing. PZ: Data curation, Formal analysis, Methodology, Writing – review & editing. YD: Data curation, Resources, Software, Visualization, Writing – review & editing. QL: Conceptualization, Data curation, Formal analysis, Methodology, Project administration, Writing – review & editing. MH: Investigation, Methodology, Project administration, Supervision, Writing – review & editing. XX: Investigation, Methodology, Resources, Writing – review & editing. QJ: Formal analysis, Investigation, Methodology, Writing – review & editing. YL: Conceptualization, Methodology, Supervision, Writing – original draft, Writing – review & editing. GL: Conceptualization, Supervision, Writing – original draft, Writing – review & editing.
